# Study on Intestinal Adaptation Mechanism of Sika Deer to High-Tannin Diet and the Mediating Role of Gut Microbiota

**DOI:** 10.3390/vetsci13101076

**Published:** 2026-10-12

**Authors:** Tianxiang Zhang, Yang Zhang, Manyu Zhang, Jun Liu, Yixuan Zhang, Yingying Feng, Weijie Han, Ziwei Zhu, Haijun Chen, Xiaofeng Huang

**Affiliations:** 1 Jiangxi Academy of Forestry, Nanchang 330032, China; 2 Key Laboratory of Wildlife Conservation, National Forestry and Grassland Administration (Jiangxi Academy of Forestry), Nanchang 330032, China; 3 Xiajiang Lishan Sika Deer Breeding Base, Ji’an 331401, China

**Keywords:** sika deer, high-tannin diet, fecal microbiota transplantation, metabolic adaptation, gene expression

## Abstract

Tannins are plant-derived polyphenolic anti-nutritional factors that impair nutrient absorption, damage the intestinal barrier, and disrupt metabolic homeostasis. Some animals, however, have an innate resistance to the effects of tannins. Sika deer (*Cervus nippon*) exhibit strong tolerance to long-term consumption of tannin-rich oak plants, and *Akkermansia muciniphila* is considered a key functional bacterium, yet the underlying mechanism of this remains unclear. In this study, we transplanted fecal microbiota from tannin-acclimated sika deer into antibiotic-pretreated C57BL/6J mice, followed by tannin gavage to establish a stress model. Growth performance, serum glycolipid indices, and hepatic metabolic gene and protein expression were detected. Results showed no significant differences in body weight, fat deposition, or serum biochemical parameters between groups. In the transplantation group, key genes involved in glycolysis, detoxification, and lipid metabolism were significantly upregulated, while *CYP7A1*, a bile acid synthesis rate-limiting gene, was significantly downregulated at both transcriptional and protein levels. *Akkermansia* was specifically enriched in the gut of transplanted mice. In conclusion, the microbiota of the sika deer gut alleviates tannin toxicity and enhances host tolerance by regulating hepatic glycolipid and bile acid metabolism pathways. The results of our study provide new insights into the adaptation mechanisms of herbivores to high-tannin diets.

## Simple Summary

Sika deer are well-adapted to consuming tannin-rich plants in the wild. However, farm-raised sika deer fed low-tannin formulated diets may lose this natural tolerance. In this study, we acclimated captive sika deer to a high-tannin diet and transferred their gut microbiota into laboratory mice which were subsequently exposed to tannins. Recipient mice receiving deer microbiota maintained normal physiological status and exhibited hepatic metabolic changes consistent with a protective response. Our work suggests that gut microbiota contribute to tannin tolerance in sika deer and may inform feeding strategies for farmed sika deer.

## 1. Introduction

Tannins are polyphenolic metabolites widely distributed among diverse plant species and tissues. Their concentrations and chemical profiles vary markedly depending on plant species, organ, developmental stage, and growing conditions [1]. Tannins are broadly classified as hydrolyzable and condensed tannins, which exert distinct biological activities and physiological effects in herbivores. As important chemical defense agents produced by plants in response to herbivory, their effects exhibit a clearly dose-dependent trend [2,3]. High concentrations of tannins result in the formation of insoluble complexes with proteins and carbohydrates [4], reducing the digestibility of nutrients [5], and damaging gastrointestinal mucosa [6]. Low-dose tannins, however, can modulate intestinal microecology and exert certain beneficial effects on host health [7,8]. The biological outcomes of tannin exposure are not merely dose-dependent; they are also modulated by the chemical structure and molecular weight of tannins, their protein-binding capacity, basal diet composition, animal species, physiological status and prior exposure to tannins.

Over the long-term course of co-evolution of plants and herbivores, herbivores have gradually evolved multiple tannin tolerance strategies, including salivary tannin-binding proteins, altered feeding behavior, modified digestive physiology, and microbial detoxification pathways [9,10,11,12]. Among them, gut microbiota, with its metabolic plasticity, can degrade toxins, regulate host metabolism, and maintain intestinal homeostasis, and has attracted attention as a research hotspot in dietary adaptation mechanisms [13,14,15].

Sika deer (*Cervus nippon*) is an endemic rare herbivore in East Asia. In the wild, sika deer naturally consume tannin-rich, oak-derived forage [16,17]. *Akkermansia muciniphila*, a key symbiotic bacterium in the intestinal mucus layer, can degrade mucin, strengthen the intestinal barrier, and regulate energy metabolism and detoxification functions. In recent years, *A. muciniphila* has been considered to be closely associated with adaptation to various dietary stresses. Our preliminary study found that the abundance of intestinal *A. muciniphila* changed significantly both in sika deer after tannin feeding and in mice receiving deer-derived microbiota transplantation, suggesting that this bacterium is a core functional bacterium for sika deer in response to high-tannin stress. Nevertheless, direct experimental evidence linking *A. muciniphila* to tannin adaptation remains limited. Therefore, in the present study, we investigated the potential association of *A. muciniphila* with host responses to a high-tannin diet.

Existing studies have mostly focused on the genetic regulation of host detoxification genes such as *UGT2B* in sika deer [18], while the *Akkermansia*-mediated microbiota–host interaction mechanism remains poorly elucidated. In addition, captive sika deer are long-term fed low-tannin artificial diets, resulting in significant differences in gut microbiota structure (including *Akkermansia*) compared with wild individuals, accompanied by decreased tannin tolerance.

Fecal microbiota transplantation (FMT) is a classic methodological approach to decipher microbiota–host interactions [19,20,21]. In this study, using captive sika deer as donors, gut microbiota (including *Akkermansia*) was induced to undergo adaptive differentiation to high-tannin feeding. The acclimated fecal microbiota was transplanted into C57BL/6J mice, which were then subjected to the same tannin stress treatment. This study aimed to investigate the effects of sika deer gut microbiota (with a focus on *Akkermansia*) on growth, fat deposition, glucose and lipid metabolism, and liver-related gene expression in mice, and to elucidate the molecular mechanism by which *Akkermansia* mediates sika deer adaptation to high-tannin diets, enriching the theory of co-evolution between herbivores and plant chemical defenses, and provide a theoretical basis for scientific feeding and healthy breeding of captive sika deer.

## 2. Materials and Methods

### 2.1. Experimental Animals and Grouping

Seven 18-month-old male captive sika deer were selected from the Xiajiang Lishan Sika Deer Farm for the dietary tannin supplementation experiment. All individuals were clinically healthy and were housed and fed under standardized conditions. All experimental subjects were housed in separate enclosures.

Ten healthy 8-week-old SPF male C57BL/6J mice were purchased from GemPharmatech Co., Ltd. (Nanjing, China; Animal Production License No. SCXK (Su) 2023-0009). Mice were housed at 20–26 °C with 40–70% relative humidity, with *ad libitum* access to standard chow and drinking water. After 3 days of adaptive feeding, all mice were given an antibiotic cocktail (ampicillin 1 g/L, neomycin sulfate 1 g/L, metronidazole 1 g/L) in drinking water for seven consecutive days to deplete native gut microbiota.

### 2.2. High-Tannin Diet Feeding of Sika Deer and Fecal Microbiota Collection

Hydrolyzable tannin (20 g, approximately 2% of the diet) was added to the daily ration of the experimental sika deer for 1 consecutive month. After feeding, using sterile gloves, fresh feces were collected into sterilized centrifuge tubes, snap-frozen in liquid nitrogen, and transported back to the laboratory for processing. Two grams of fresh sika deer feces were ground and dissolved in 4 mL of 0.1 M PBS (pH 7.2), fully homogenized, and centrifuged at low speed. The supernatant was collected and stored at low temperature.

### 2.3. Fecal Metabolomics Analysis of Sika Deer Before and After High-Tannin Feeding

Fecal samples were collected from sika deer before and after the one-month-long period of tannin feeding. After protein precipitation with methanol–acetonitrile and centrifugation, the supernatant was collected for untargeted metabolomics analysis using UPLC-MS/MS. Raw data were processed with Progenesis QIVersion 2.0 (Waters, Milford, MA, USA) for peak alignment, retention time correction, and peak intensity normalization to obtain a metabolite quantification matrix.

For metabolite extraction, 1 g of fecal sample was placed in an EP tube, and 1000 μL of 80% aqueous methanol solution was added. The mixture was vortexed thoroughly and incubated on ice for 5 min, followed by centrifugation at 15,000× *g*, 4 °C for 15 min. The supernatant was transferred to a new tube and centrifuged again at 15,000× *g*, 4 °C for 20 min. The resulting supernatant was collected and freeze-dried. The dried residue was reconstituted with an appropriate volume of 10% methanol solution based on the original sample volume, and the reconstituted solution was subjected to UPLC-MS/MS analysis. Quality control (QC) samples were generated by pooling equal-volume aliquots from each experimental sample. QC samples were injected repeatedly throughout the analytical run to monitor instrument performance, assess the stability of the chromatographic-mass spectrometric system, and support data quality control. Blank samples were prepared using 53% aqueous methanol solution in place of biological material; the blank samples were subjected to the same sample-preparation procedures as the experimental samples. Chromatographic peak picking and integration were performed using XCMS software (version 3.16.0). The peak area of each feature was used as a relative quantitative measure for the corresponding metabolite. Background ions were removed using signals acquired from blank samples. Raw quantitative results were normalized by total peak area. Metabolites with a coefficient of variation (CV) < 30% among QC samples were retained for downstream analysis. Metabolite annotation and relative quantitative datasets were obtained after these filtering steps. UPLC-MS/MS analysis was performed on a commercial platform in both positive- and negative-ion electrospray ionization modes. Metabolite identification was conducted by matching accurate mass-to-charge ratios and fragment spectra against public metabolite databases.

After data preprocessing, partial least-squares-discriminant analysis (PLS-DA) was applied to evaluate global metabolic profile differences. Orthogonal partial least squares discriminant analysis (OPLS-DA) was further used for screening differential metabolites, with screening criteria of VIP > 1.0, *p* < 0.05, and |log_2_FC| > 1. KEGG pathway enrichment analysis was performed on the differential metabolites. Multivariate statistical analyses including PLS-DA and OPLS-DA were performed on the filtered metabolomics dataset. The OPLS-DA model was validated by 1000-fold permutation testing to avoid overfitting (Supplementary Figure S1). Multiple-testing correction was applied for screening of differential metabolites.

### 2.4. Mouse FMT Experiment

Fecal samples were collected from seven tannin-acclimated sika deer for individual microbiota sequencing. For FMT inoculum preparation, a sufficient amount of fresh feces (>20 g) obtained from one selected sika deer donor was used. Fresh feces (2 g) were ground and suspended in 4 mL of sterile PBS. The mixture was thoroughly vortexed, followed by low-speed centrifugation (2000× *g*, 4 °C, 10 min). The supernatant was collected and kept at low temperature for subsequent intragastric administration.

Prior to FMT, mice received an antibiotic cocktail to deplete their indigenous gut microbiota. Five mice in the experimental group were subjected to daily gavage with 200 μL of sika deer gut microbiota suspension, while five mice in the control group were gavaged with 200 μL of sterile 0.1 M PBS (pH 7.2). Fresh mouse fecal samples were collected for 16S rRNA amplicon sequencing. Sequencing library construction and bioinformatic processing were performed using standard amplicon-sequencing workflows. PICRUSt2 (phylogenetic investigation of communities by reconstruction of unobserved states) was used to infer microbial functional profiles based on 16S rRNA gene data. PICRUSt2 generates computationally predicted functional profiles, which do not represent directly measured in situ microbial functions.

### 2.5. Tannin Gavage Treatment in Mice

Food-grade tannic acid (hydrolyzable tannin, Shanghai Xintai Industrial Co., Ltd. (Shanghai, China)) complying with the Chinese national standard GB 1886.303-2021 (mass fraction ≥96%) was used. The batch used in this study had a manufacturer-declared tannic acid content of 98%. Tannic acid from the same batch was used for both sika deer feeding and mouse gavage treatment. Tannic acid was dissolved in sterile normal saline to prepare a working solution containing 10 mg/0.1 mL, and sonication and heating were used to facilitate complete dissolution. Considering the approximate LD_50_ of tannic acid (~1800 mg/kg body weight) and our preliminary tests, in which 20 mg per mouse resulted in acute lethal toxicity, 10 mg of tannic acid per mouse per day was selected as a sublethal tannin challenge dose. Given an average mouse body weight of 20 g, this gavage dose corresponded to 500 mg tannic acid per kg body weight. Following fecal microbiota transplantation, mice in both groups received tannic acid solution by daily intragastric gavage for 20 consecutive days. The tannic-acid dose, treatment duration, and administration procedure were kept identical between the control and FMT groups.

The commercial food-grade tannic acid met GB 1886.303-2021 with a manufacturer-declared tannic acid content of >98%, although its actual purity was not independently verified in-house.

### 2.6. Sample Collection and Processing

All samples were collected 24 h after the final intragastric administration of tannic acid. After tannin treatment, mice were weighed. Mesenteric fat, perirenal fat, inguinal subcutaneous white adipose tissue, and dorsal brown adipose tissue were dissected and weighed. Blood was collected by cardiac puncture, and serum was separated. A fully automatic biochemical analyzer (BK-600) Implen GmbH, Munich, Germany. was used to measure blood glucose (BG), triglycerides (TGs), total cholesterol (CHO), high-density lipoprotein cholesterol (HDL-C), and low-density lipoprotein cholesterol (LDL-C). Consistent with the sika deer sampling strategy, fresh mouse feces were collected for gut microbiota composition analysis. Liver tissues were snap-frozen in liquid nitrogen and stored at −80 °C for gene expression detection. Intestinal contents were then collected for microbiota composition analysis.

Liver tissues were dissected post-mortem. Partial liver tissue was fixed for immunohistochemical staining, and the remaining tissue was snap-frozen in liquid nitrogen for RNA extraction and gene expression detection. Owing to sample consumption for IHC preparation, the sample size for hepatic mRNA qPCR detection was *n* = 3 per group, whereas other phenotypic and sequencing analyses were performed with *n* = 5 per group.

### 2.7. Gene Expression Detection

Total RNA was extracted from mouse liver tissue using Trizol reagent (CW0580S, CWBIO), and mRNA was purified using an RNA ultra-pure extraction kit (CW0581M, CWBIO). The concentration and purity (OD_260_/OD_280_) of mRNA were determined using an ultraviolet-visible spectrophotometer (NP80, NanoPhotometer, Implen GmbH, Munich, Germany). cDNA was synthesized using an RNA reverse transcription kit (R223-01, Vazyme). Quantitative real-time PCR was performed on a CFX Connect™ Real-Time PCR System (Bio-Rad, Shanghai, China).

The reaction procedure was as follows: pre-denaturation at 95 °C for 30 s; denaturation at 95 °C for 10 s, annealing at 58 °C for 30 s, extension at 72 °C for 30 s, for 40 cycles. *β-actin* was used as the internal reference gene, and the relative expression of target genes was calculated using the 2^−ΔΔCt^ method. Primer sequences are shown in Table S1.

### 2.8. Immunohistochemical Assay

The immunohistochemical SP method was used to detect the localization and expression levels of target proteins in mouse liver and small intestine tissues, including gluconeogenesis-related proteins (GPI, PFKL, PKM2, GLUT1) and lipid metabolism-related proteins (ACC1, SREBP-1c, PPAR-γ, FAS, CYP7A1), to verify the reliability of qPCR results.

Information on all antibodies used for immunohistochemistry in this study is listed in Table S2.

Aperio ImageScope (version v12.3.3.5048.) was used for grayscale analysis of positive regions. The average grayscale value represented the relative expression level of the target protein. Five random fields per sample were selected for statistical analysis.

### 2.9. Data Analysis

All experimental data were analyzed using SPSS 26.0 (IBM Corp., Armonk, NY, USA). Measurement data are expressed as mean ± standard deviation ($\bar{x}$ ± s). For two-group comparisons between control and FMT-transplanted mice, an independent-sample *t*-test was used, and effect sizes and 95% confidence intervals were calculated and reported. One-way analysis of variance (ANOVA) followed by Duncan’s new multiple-range test was used only for multigroup comparisons of sika deer data.

The qPCR gene-expression data, immunohistochemical relative protein abundance data, and metabolism-related phenotypic data were tested for normality and homogeneity of variance. The nonparametric Mann–Whitney U test was used when datasets did not meet the assumption of normality. Pearson correlation analysis was performed to explore associations between target gene and protein levels and metabolic indicators; correlation results were interpreted cautiously given the limited biological sample sizes (*n* = 3–5). Individual biological replicate data points are shown in Supplementary Figures S2–S4.

For hepatic qPCR assays, *n* = 5 per group for glucose metabolism-related genes; due to depletion of RNA samples during supplementary experiments, *n* = 3 per group for lipid -metabolism-related genes. For other measurements, including body weight, adipose tissue weight, serum biochemistry, and 16S rRNA sequencing, *n* = 5 per group.

### 2.10. AI Disclosure Statement

No generative artificial intelligence (GenAI) tools were used in the preparation of this manuscript. No AI was applied for manuscript writing, figure generation, data production, experimental design, data collection, data analysis, or interpretation of results. All work described in this study was performed solely by the authors of this paper.

## 3. Results

### 3.1. Differences in Fecal Metabolome of Sika Deer After High-Tannin Diet Feeding

Partial least-squares-discriminant analysis (PLS-DA) showed that fecal metabolic profiles before (Group B) and after (Group A) tannin feeding were clearly separated in the principal component space (Figure 1). Samples from the two groups were clustered in distinct regions with good intra-group reproducibility (Component 1 explained 33.7% of variance, while Component 2 explained 24.5%), indicating that high-tannin feeding significantly altered the hindgut metabolic microenvironment of sika deer.

Based on the OPLS-DA model, a total of 28 significantly differentially expressed metabolites were identified (VIP > 1.0, *p* < 0.05, |log_2_FC| > 1), among which only 2 metabolites were significantly upregulated after feeding, while the remaining 26 were significantly downregulated (Figure 2).

The upregulated metabolites were 4-(β-D-glucosyloxy)-3-hydroxybenzoic acid and Trungapeptin C. The former is a phenolic acid glycoside, the final product of tannin/polyphenol detoxification mediated by hepatic UGT2B via glycosylation. The latter is a cyclic peptide involved in intestinal homeostasis regulation.

Downregulated metabolites primarily included terpenoids, alkaloids, bile acids, amino acids/peptides, and SCFA precursors. Among them, the isoquinoline alkaloid Radicamine B showed the most notable decrease, suggesting that under tannin stress, sika deer actively suppress non-essential secondary metabolic pathways to reduce energy consumption and prioritize metabolic resources for detoxification and energy supply.

### 3.2. Verification of Mouse Fecal Microbiota Transplantation Efficiency

After the transplantation procedure, 16S rRNA sequencing was performed on fecal samples from donor sika deer (D), transplantation group mice (T), and control group mice (C). The Venn diagram results are shown in Figure 3.

To clarify whether sika deer fecal microbiota successfully colonized the recipient mouse intestine and reshaped the gut microbial community structure, 16S rRNA high-throughput sequencing was carried out on fecal samples from sika deer donors (D), control mice (C), and sika deer FMT mice (T). Transplantation efficiency was verified on three aspects: shared OTUs, overall community structure, and relative abundance of dominant species.

Venn diagram results (Figure 3) showed that a total of 3301 OTUs were detected across the three groups. There were 966 unique OTUs in sika deer donors, 894 unique OTUs in control mice, and 291 unique OTUs in transplantation mice. A total of 992 OTUs were shared by all three groups. The transplantation group harbored unique microbiota from both sika deer donors and control mice, demonstrating that cross-species FMT achieved the transfer of deer-derived microbiota to the mouse intestine. However, a large number of sika deer-specific OTUs were not able to stably colonize mice, indicative of an obvious cross-species microbial colonization barrier.

PCoA based on a Bray–Curtis distance matrix was performed, and PerMANOVA was used to test community differences among groups (Figure 4). PC1 explained 59.769% of the microbial variance. The three groups showed clear separation in the two-dimensional space: sika deer donors (D) clustered independently on one side, while control mice (C) and transplantation mice (T) partially overlapped. PerMANOVA results showed df = 2, R^2^ = 0.7582, *p*-value = 0.001, indicative of extremely significant differences in overall gut microbiota structure among the three groups, and FMT significantly altered the original gut microbial composition of mice.

The relative abundance of the top 10 dominant families (Figure 5) showed that sika deer donors were enriched with a large number of deer-specific fiber-degrading bacteria. The microbiota structure of transplantation mice partially shifted toward sika deer donors but still retained the characteristics of mouse native symbiotic bacteria.

Further multiple comparisons were performed on significantly differentially abundant key genera (Figure 6). Letters represent inter-group differences (different letters indicate *p* < 0.05, same letters indicate no significant difference).

*Akkermansia*: Both the control group (C) and sika deer donor (D) had extremely low abundance and were marked as “a”. The transplantation group (T) showed significantly increased abundance, marked as “b”, serving as the core genus specifically enriched after transplantation.

*Alistipes* exhibited high abundance in both control and sika-deer-donor groups and decreased significantly in FMT mice.

*CAG-873* (uncultured genus within the family Bacteroidaceae): Significantly enriched only in control mice (b), but significantly decreased in sika deer donors (D) and transplantation mice (T) (a). *CAG-873* is a mouse-host-specific symbiotic bacterium that cannot maintain its original abundance after cross-species transplantation.

*Treponema*, *Xylanibacter*, *UCG-005*: High abundance only in sika deer donors (D) (b), barely detectable in both C and T groups (a). These are deer-specific bacteria for crude fiber digestion and cannot colonize the mouse intestine.

*Lactobacillus*, *Muribaculum*: Significantly lower abundance in sika deer donors (D) (a). The transplantation group (T) recovered to a level that was not significantly different from the control group (C) (b), indicating that transplantation restored the homeostasis of mouse basal symbiotic bacteria.

*Lachnospiraceae_NK4A136_group*: High abundance in the control group (C) (b), low abundance in the donor (D) (a), and marked “ab” in the transplantation group (T). The abundance of this group was intermediate between the two groups, with a partial shift toward the donor but no statistically significant difference.

These results indicate that sika deer FMT can successfully reshape the gut microbiota of tannin-stressed mice, with specific enrichment of *A. muciniphila*. However, sika deer-specific crude fiber-degrading bacteria face cross-species colonization barriers, and the mouse-native bacterium *CAG-873* was significantly downregulated after transplantation. Altered microbiota composition serves as an important upstream signal regulating host liver metabolism.

### 3.3. Differences in Serum Physiological Indices and Body Fat Parameters in Mice

After tannin treatment, various adipose tissues were dissected and weighed. To detect fasting blood glucose and serum lipid indices, cardiac blood was collected. Body weight, fat deposition, and serum glycolipid biochemistry between the control and sika deer FMT groups were statistically compared (Figure 7).

Body fat results showed no significant differences in final body weight, mesenteric fat, perirenal fat, inguinal white adipose tissue, or dorsal brown adipose tissue weight between the transplantation group and the control group (*p* > 0.05, *n* = 5), indicating that sika deer-derived adaptive microbiota can maintain mouse fat deposition homeostasis under continuous tannin stress, and the anti-nutritional and lipid metabolism-interfering effects of tannins are alleviated.

Serum glycolipid biochemical results showed that fasting blood glucose and low-density lipoprotein cholesterol (LDL-C) in the transplantation group showed a mild increasing trend compared with the control group, while total cholesterol (TC), triglycerides (TGs), and high-density lipoprotein cholesterol (HDL-C) showed a slight decreasing trend. However, none of these serum indices reached statistical significance between the two groups (*p* > 0.05, *n* = 5).

Taken together, body fat and serum biochemical results indicate that sika deer gut microbiota does not cause severe glycolipid metabolic disorders in mice under tannin stress, but only causes slight fluctuations in blood glucose and blood lipids. Microbiota forms a compensatory balance by regulating hepatic glycolipid metabolic pathways, maintaining host energy and lipid homeostasis, and alleviating the inhibitory effects of tannins on nutrient absorption and lipid metabolism.

### 3.4. Differences in Hepatic Gene Expression Between the Two Groups

Quantitative real-time PCR was used to detect the mRNA relative expression levels of key genes involved in glucose metabolism, lipid metabolism, bile acid synthesis, and detoxification pathways in mouse liver. *β-actin* was used as the internal reference gene, and relative expression levels were calculated using the 2^−ΔΔCt^ method (Figure 8).

Key glycolytic genes *GPI*, *PFKL*, *ALDOA*, *PGK*, *GAPDH*, and *PKM2* were all significantly upregulated in the liver of the transplantation group (*p* < 0.05, *n* = 5). The glucuronide detoxification gene *UGT2B* was significantly higher than that in the control group (*p* < 0.01, *n* = 5). As a polyphenolic toxin, tannins require UGT2B-mediated glucuronidation to form water-soluble glycoside products for excretion. The substantial upregulation of *UGT2B* in the transplantation group demonstrates that deer-derived microbiota significantly enhances hepatic tannin detoxification capacity in mice. Simultaneously, the synchronous activation of all glycolytic genes suggests that the microbiota induces enhanced glycolytic flux in the liver, compensating for insufficient nutrient absorption caused by tannins and providing sufficient acetyl-CoA carbon sources for the host.

The de novo lipogenic transcription factor *SREBP-1c*, key synthase *FAS*, and lipid metabolism regulatory nuclear receptor *PPARγ* were significantly upregulated in the transplantation group liver (*p* < 0.01, *n* = 3). Lipolytic genes *TGH* and *HSL* were also significantly increased (*p* < 0.01, *n* = 3), indicating that both hepatic lipogenesis and lipolysis pathways are synergistically activated after transplantation, and lipid metabolism maintains a dynamic turnover balance to avoid abnormal lipid accumulation or excessive lipolysis.

The rate-limiting bile acid synthesis gene *CYP7A1* was significantly downregulated in the transplantation group (*p* < 0.01, *n* = 3), suggesting that the transplanted microbiota negatively feedback inhibits hepatic de novo bile acid synthesis via intestinal signals. Decreased *CYP7A1* expression reduces the shunting of cholesterol and acetyl-CoA toward bile acids, allocating more carbon sources to glycolysis and fatty acid synthesis, thus achieving hepatic carbon source redistribution. Notably, lipid-metabolism-related qPCR analyses were performed with *n* = 3 per group, and the reduced sample size may limit statistical robustness for these gene expression results.

The overall gene expression pattern shows that adaptive FMT from sika deer can synchronously activate hepatic glycolysis, detoxification, lipogenesis, and lipolysis pathways while inhibiting the bile acid synthesis pathway under tannin stress. Through coordinated regulation of multiple metabolic pathways, host tannin tolerance is improved, and glycolipid metabolic homeostasis is maintained, providing a transcriptional basis for subsequent immunohistochemical protein quantification.

### 3.5. Differences in Hepatic Immunohistochemical Results Between the Two Groups

Liver tissues from each group were fixed, dehydrated, embedded, and sectioned for immunohistochemical staining. Detailed information on primary antibodies is provided in the Supplementary Tables. Sections were observed under a 10× objective lens, and 3 non-overlapping fields were randomly selected per sample for imaging. Aperio ImageScope was used for quantitative image analysis in order to calculate the average optical density (Iavg/AOD) (Figure 9).

Iavg is defined as the ratio of total integrated optical density of all positive pixels to the total number of positive pixels. This dimensionless relative value reflects the average expression abundance of the target protein in hepatocytes. Quantitative statistical results showed that compared with the control group, the average optical density of hepatic CYP7A1 protein was significantly decreased in the experimental group, while the average optical densities of PPARγ and PFKL proteins were significantly increased, all with statistically significant inter-group differences (*p* < 0.05). It should also be noted that immunohistochemical average optical density represents a semiquantitative readout; variations in tissue section quality and staining background may introduce technical bias.

## 4. Discussion

Tannins are polyphenolic anti-nutrients that bind proteins and polysaccharides [22], impair intestinal nutrient digestion [9], and damage the gut mucosa [23], constituting major dietary stressors for herbivores [24,25]. Sika deer can sustain normal growth and reproduction even with long-term intake of tannin-rich oak foliage. Previous studies primarily focused on host detoxification genes such as *UGT2B* while neglecting the compensatory functions of hindgut microbiota [18,26]. Captive sika deer fed low-tannin artificial diets exhibit drastically altered gut microbiota and reduced tannin tolerance [27,28]. In the present work, we established a cross-species FMT mouse model using a fecal inoculum from tannin-adapted sika deer. By integrating 16S rRNA amplicon sequencing, fecal metabolomics, hepatic qPCR, and immunohistochemistry, this study provides correlative evidence regarding the potential mechanisms by which sika deer hindgut microbiota may modulate host responses to tannin exposure. Our findings provide insights into herbivore adaptation to plant secondary metabolites and may inform targeted microbial feeding strategies in farmed sika deer [26].

Limited by sampling constraints, we collected only fecal samples to characterize hindgut microbiota, and eschewed the collection of rumen microbes [29,30,31]. The rumen is considered the primary site for microbial tannin degradation in ruminants. Though fecal profiles do not fully represent rumen communities and cannot capture ruminal tannin-degrading activity, they can partially reflect cecal and colonic microbial composition and functions relevant to post-ruminal tannin metabolism. Therefore, our interpretation is limited to hindgut microbiota–host interactions, and caution is warranted when extrapolating our findings to overall rumen-mediated tannin adaptation in sika deer. An antibiotic cocktail was administered to deplete indigenous gut microbiota. We did not perform amplicon sequencing to independently verify the extent of native microbiota depletion, which represents a limitation of this study. Our 16S sequencing revealed prominent cross-species colonization barriers: deer-specific fiber-degrading genera *Treponema*, *Xylanibacter,* and *UCG-005* failed to colonize mouse intestines, whereas the mouse-native symbiont *CAG-873* was depleted post-FMT (Figure 6). *CAG-873* secretes bile salt hydrolase to activate intestinal FXR signaling and upregulate hepatic *CYP7A1* [32,33,34]. Nevertheless, both *CYP7A1* mRNA and protein were significantly downregulated in FMT mice, indicating that the newly enriched *A. muciniphila* correlated with alterations in hepatic CYP7A1 expression. Meanwhile, Lactobacillus and Muribaculum, which were scarce in deer donors, recovered to baseline levels in recipient mice, suggesting deer-derived microbiota restore core symbiosis and alleviate tannin-induced intestinal dysbiosis. In addition, the relative abundance of A. muciniphila was determined solely by 16S rRNA amplicon sequencing; we did not perform independent species-specific qPCR for absolute quantification of *A. muciniphila*.

For ruminants, a three-tiered tannin utilization strategy operates sequentially in the rumen, hindgut, and liver [35]. Macromolecular tannins are first degraded into small phenolic compounds by ruminal tannase [8,9]; hindgut microbes may further transform residual tannin fragments into phenolic acid derivatives, although our dataset cannot identify which specific taxa mediate this biotransformation. Fecal metabolomics identified 28 differential metabolites in tannin-treated sika deer. Only two metabolites were upregulated: the tannin detoxification end-product 4-(β-D-glucosyloxy)-3-hydroxybenzoic acid and homeostasis-modulating peptide Trungapeptin C [36]. The remaining 26 metabolites, including SCFA precursors, bile acids, and lipids, were suppressed, with the alkaloid Radicamine B showing the sharpest decline (Figure 2). These data suggest that tannin stress may trigger a global redistribution of metabolic resources. One plausible scenario is that the microbiota prioritize tannin transformation to generate absorbable phenols, while enhanced intestinal bile acid metabolism could alleviate the hepatic synthetic burden, and non-essential secondary metabolism is repressed to instead channel energy toward intestinal and hepatic detoxification [37]. It should be noted that this mechanistic sequence remains hypothetical and requires further experimental validation. Phenolic compounds absorbed via the portal vein undergo *UGT2B*-mediated glucuronidation to form water-soluble conjugates for excretion, completing systemic tannin detoxification [38].

In mouse tannin-challenged models, final body weight and all adipose tissue weights showed no intergroup differences, while serum glucose and lipid levels fluctuated only mildly, without exhibiting statistical significance (Figure 7). It is hypothesized that control mice lacking tannin-degrading microbiota may experience malabsorption, intestinal barrier perturbation, and hepatic metabolic stress under tannin challenge, although these phenotypes were not directly assessed in our experiment. By contrast, FMT-recipient mice exhibited coordinated hepatic adaptive responses, which may be driven by metabolites transformed by deer-derived microbiota and subsequently used as carbon substrates: all glycolytic genes and the detoxification gene *UGT2B* were markedly upregulated to boost carbon supply and tannin clearance [39]; lipogenic (*SREBP-1c*, *FAS*, *PPARγ*) and lipolytic (*TGH*, *HSL*) pathways were simultaneously activated to maintain balanced lipid turnover [40]; the rate-limiting bile acid synthetic gene *CYP7A1* was downregulated to redirect acetyl-CoA toward glycolysis and lipogenesis (Figure 8). Such coordinated hepatic reprogramming represents a protective metabolic response rather than a pathological disorder, serving as the core molecular basis of microbiota-conferred tannin resistance. In the hepatic qPCR analysis, additional lipid metabolism-related genes were included after the initial experiments. RNA samples from two mice in each group had already been exhausted, leading to a reduced number of biological replicates (*n* = 3 per group) for lipid-metabolism-related genes, whereas glucose-metabolism-related genes retained *n* = 5 per group. This reduction in sample size may reduce statistical power for lipid metabolism gene comparisons.

Immunohistochemistry further validated transcriptional results at the translational level via average optical density (Iavg) quantification (Figure 10). In qPCR data, FMT mice exhibited significantly lower hepatic *CYP7A1* protein abundance and elevated *PFKL* and *PPARγ* protein levels. Synchronous transcriptional and translational alterations provide further support that sika deer FMT reshapes hepatic carbon allocation by modulating bile acid, glycolytic, and lipogenic signaling through gut microbial cues [41,42].

While our FMT mouse model investigating gut–liver crosstalk still faces inherent cross-species limitations. Deer-specific fiber degraders are unable to stably colonize mice, and native mouse *CAG-873* is depleted; only *A. muciniphila* proliferates, and incomplete colonization partially weakens host tolerance capacity. Even so, colonizable deer-derived microbes remodel intestinal signaling and trigger coordinated activation of hepatic glycolysis, lipogenesis, lipolysis, and detoxification while suppressing *CYP7A1*, ultimately mitigating tannin-mediated intestinal and metabolic damage [43,44].

Numerous FMT studies using mice as model organisms have proven that gut microbiota exert decisive control over hepatic glycolipid and bile acid metabolism via gut–liver communication [45,46]. Under high-fat or phytotoxin stress, donor microbiota reshapes host transcriptional and protein profiles of *CYP7A1* and glycolipid genes [47]. Cross-species ruminant-to-mouse transplantation experiments also observed colonization defects of ruminant-specific fiber degraders, consistent with our findings [48,49]. Functional assays further suggested that *A. muciniphila* activates the intestinal FXR-FGF15 cascade to inhibit hepatic CYP7A1 expression. Although our observations are consistent with this potential regulatory axis, we did not directly measure intestinal FXR and FGF15 expression in the current study. Therefore, we cannot confirm that this exact pathway was activated in our FMT mouse model [50,51,52]. Unlike single-strain supplementation trials, this study used intact tannin-adapted deer microbiota to recapitulate holistic microbial compensatory responses under wild dietary pressure and enrich theories of herbivore tannin adaptation [17].

Collectively, our multi-omics, qPCR, and immunohistochemical analyses reveal correlative associations along the gut–liver axis that help explain how sika deer microbiota may modulate host responses to tannin exposure, integrating evidence from microbiota composition, gene transcription, and protein expression. This work complements previous host-gene-focused investigations and provides new insights into the mechanisms underlying sika deer adaptation to tannin-rich forage. Future work should optimize cross-species FMT protocols to improve the colonization efficiency of deer-derived microbial strains. Multi-tissue transcriptomic profiling of the liver, small intestine, and colon will help characterize the full landscape of gut–liver regulatory networks and identify previously uncharacterized molecular targets involved in tannin adaptation, further advancing our understanding of microbe–host co-adaptation to dietary stress in herbivores.

### Limitations

Several important limitations of this study should be acknowledged. First, the FMT inoculum was prepared using feces collected from only one tannin-acclimated sika deer donor, and inter-individual variation in deer gut microbiota was not evaluated in the transplantation experiment. Second, this cross-species FMT mouse model cannot fully recapitulate the native gut environment of sika deer, and the colonization capacity of deer-derived microbes may differ substantially between mice and their native deer host. Third, 16S rRNA amplicon sequencing has limited taxonomic resolution and often fails to discriminate between closely related species; therefore, species-level identification of gut bacteria cannot be guaranteed. Fourth, PICRUSt2 was used to infer microbial functional profiles from amplicon data, and these predicted functions are not equivalent to directly measured microbial metabolic activities. Fifth, hepatic qPCR analysis for lipid -metabolism-related genes was limited by reduced biological replicates (*n* = 3 per group), while glucose-metabolism-related genes retained *n* = 5 per group; this smaller sample size may reduce statistical power for lipid metabolism-related gene comparisons. Sixth, we did not perform direct manipulation experiments involving *A. muciniphila*, so the causal role of this bacterium cannot be confirmed. Seventh, we lacked direct measurements of tannin degradation activity and intestinal barrier function in the current experiment. Although we observed enrichment of *A. muciniphila* after FMT, these limitations mean that we cannot definitively identify *A. muciniphila* as the core functional strain responsible for the observed metabolic changes. Furthermore, incomplete microbial colonization in this cross-species model limits the extrapolation of mouse-derived findings to the physiological conditions of wild sika deer. We cannot fully attribute the observed hepatic phenotypes to the complete sika-deer gut microbiota; these phenotypic changes may instead reflect the effects of successfully colonized microbial taxa, including *A. muciniphila*.

## 5. Conclusions

In summary, our fecal microbiota transplantation experiment indicates that gut microbiota derived from tannin-acclimated sika deer can modulate hepatic metabolic responses to tannin exposure in recipient mice. *Akkermansia muciniphila* was markedly enriched after transplantation and was associated with host metabolic adaptations to tannin challenge, although its causal contribution remains unproven in the present study. Our findings highlight potential links between gut commensal bacteria and host responses to high-tannin dietary stress. Further animal intervention studies using a single strain are required to clarify the specific functional role of *A. muciniphila* in tannin adaptation.

## Figures and Tables

**Figure 1:**
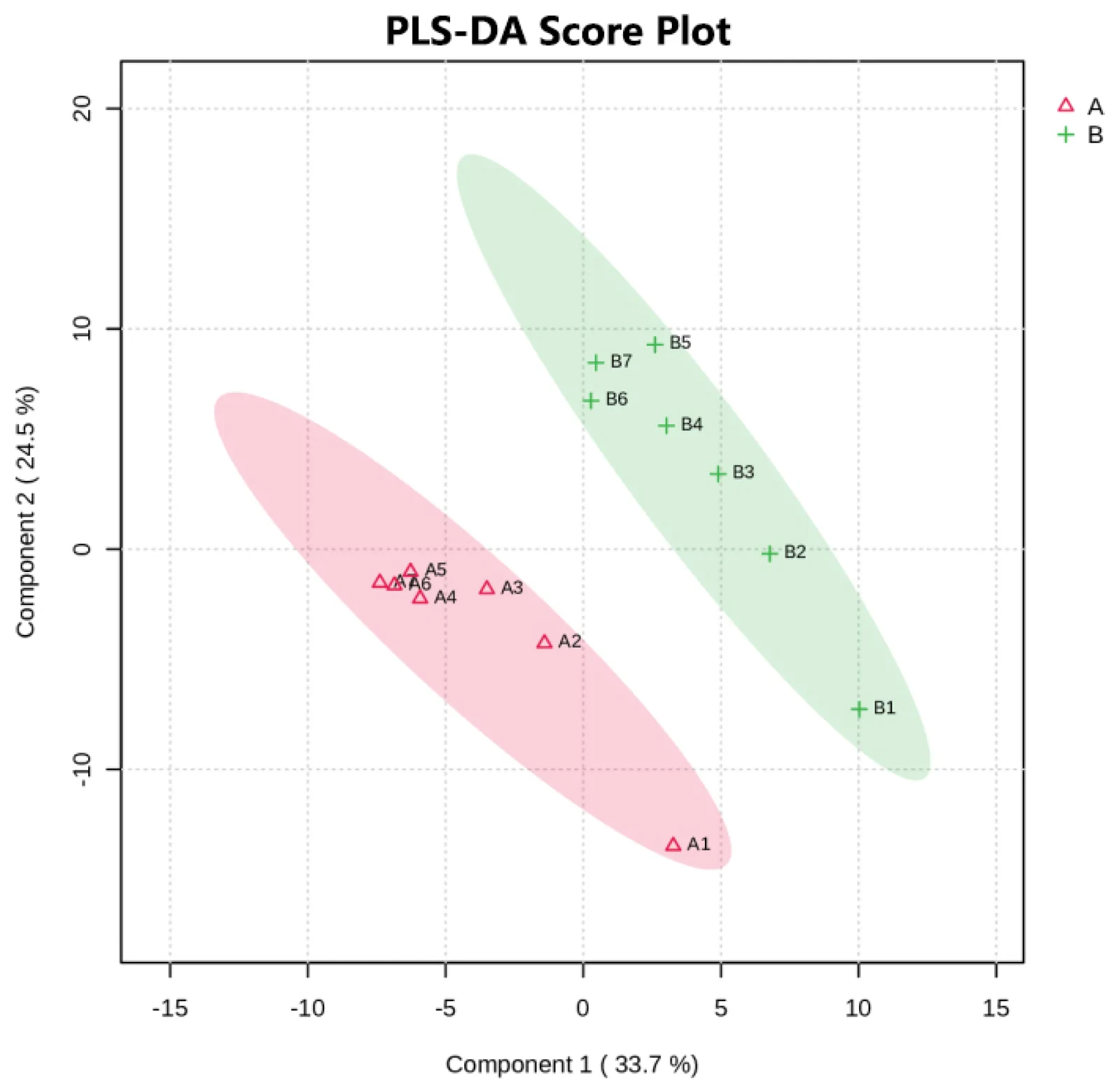
PLS-DA scores plot of sika deer fecal metabolome. A: After tannin diet group; B: before tannin diet group. Ellipses represent 95% confidence intervals. Component 1 and Component 2 denote first and second latent variables, with percentage values indicating explained variance.

**Figure 2:**
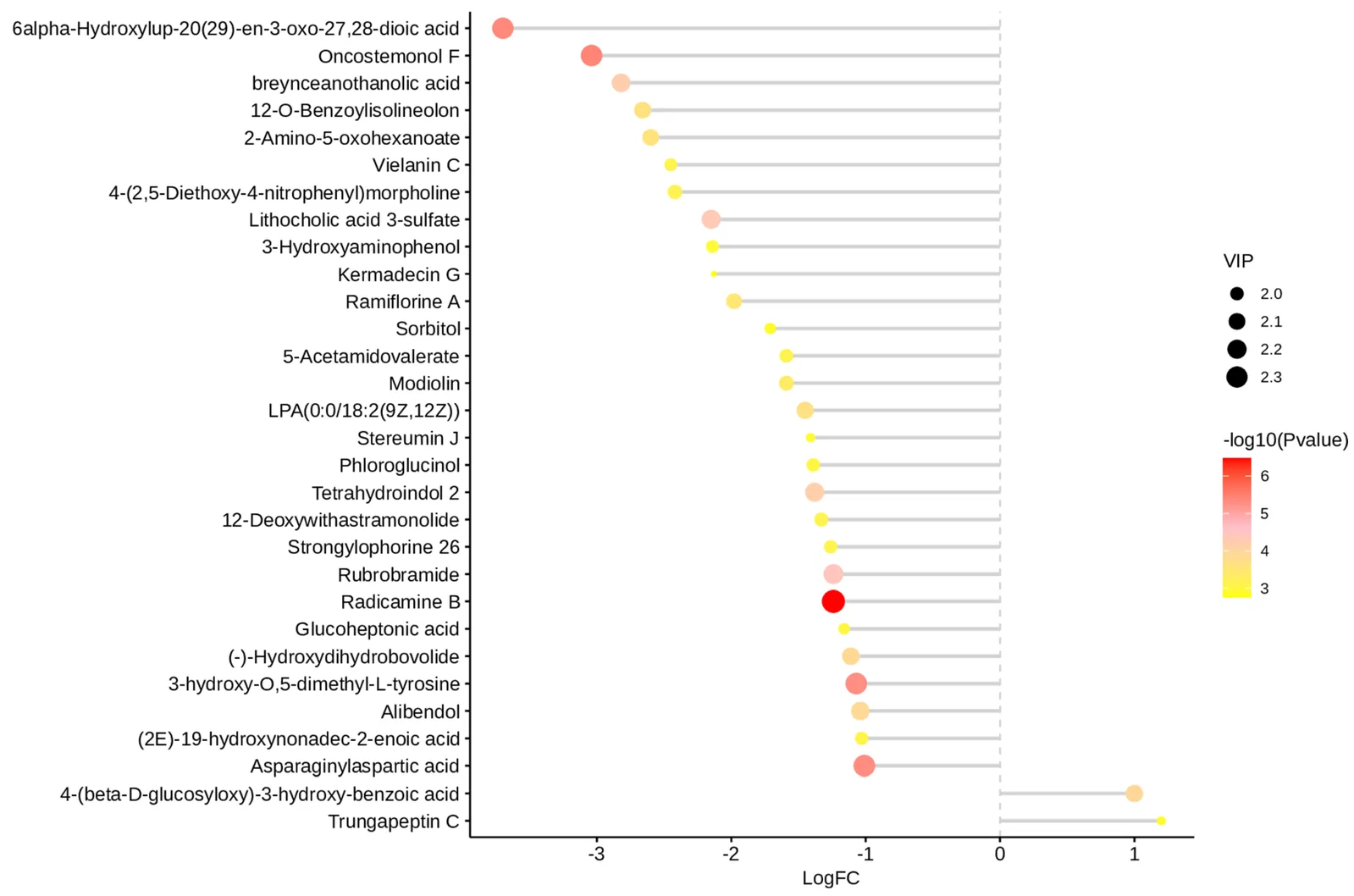
Matchstick plot of fecal metabolome in sika deer before and after tannin feeding.

**Figure 3:**
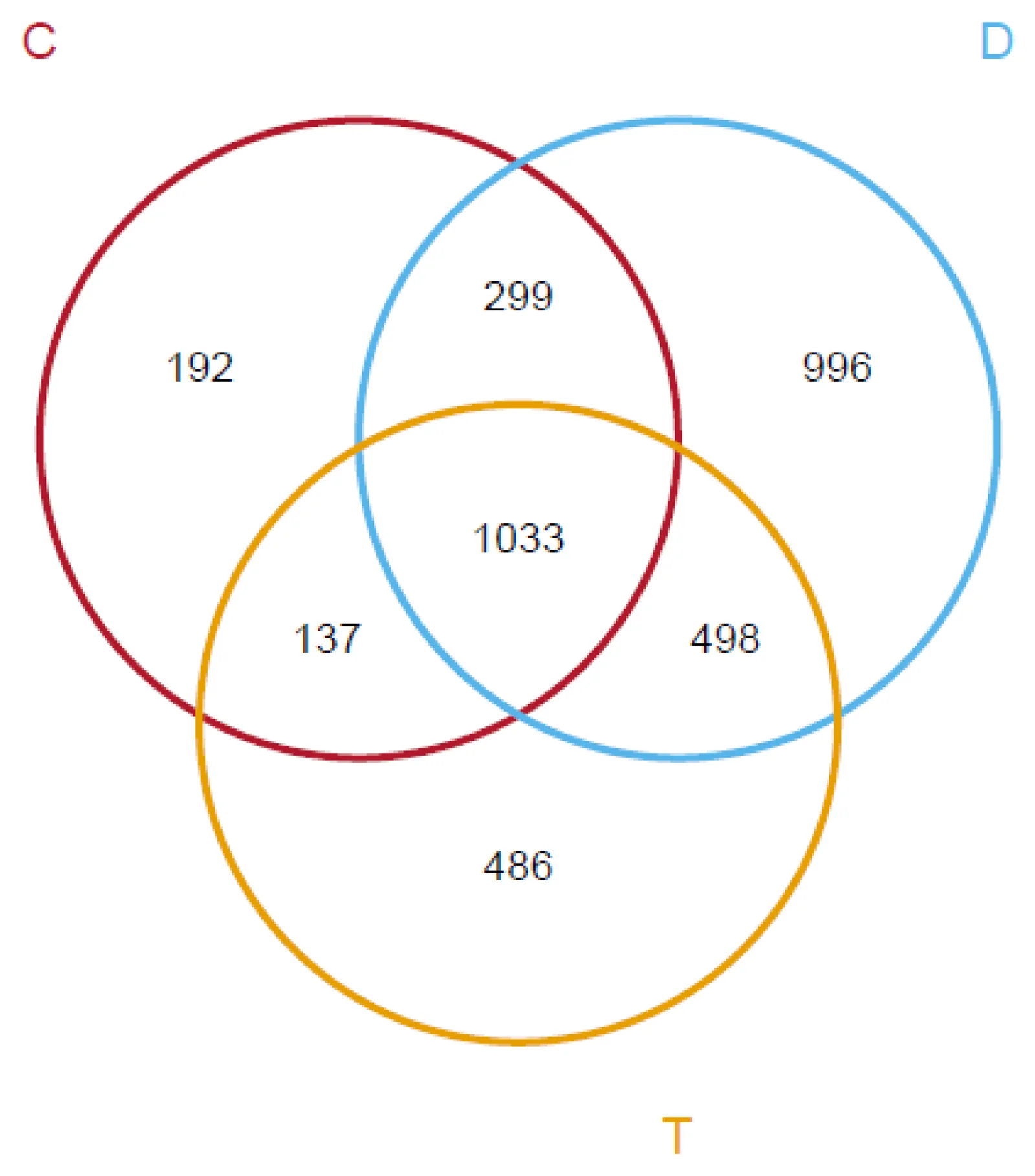
Venn diagram comparison among the three groups.

**Figure 4:**
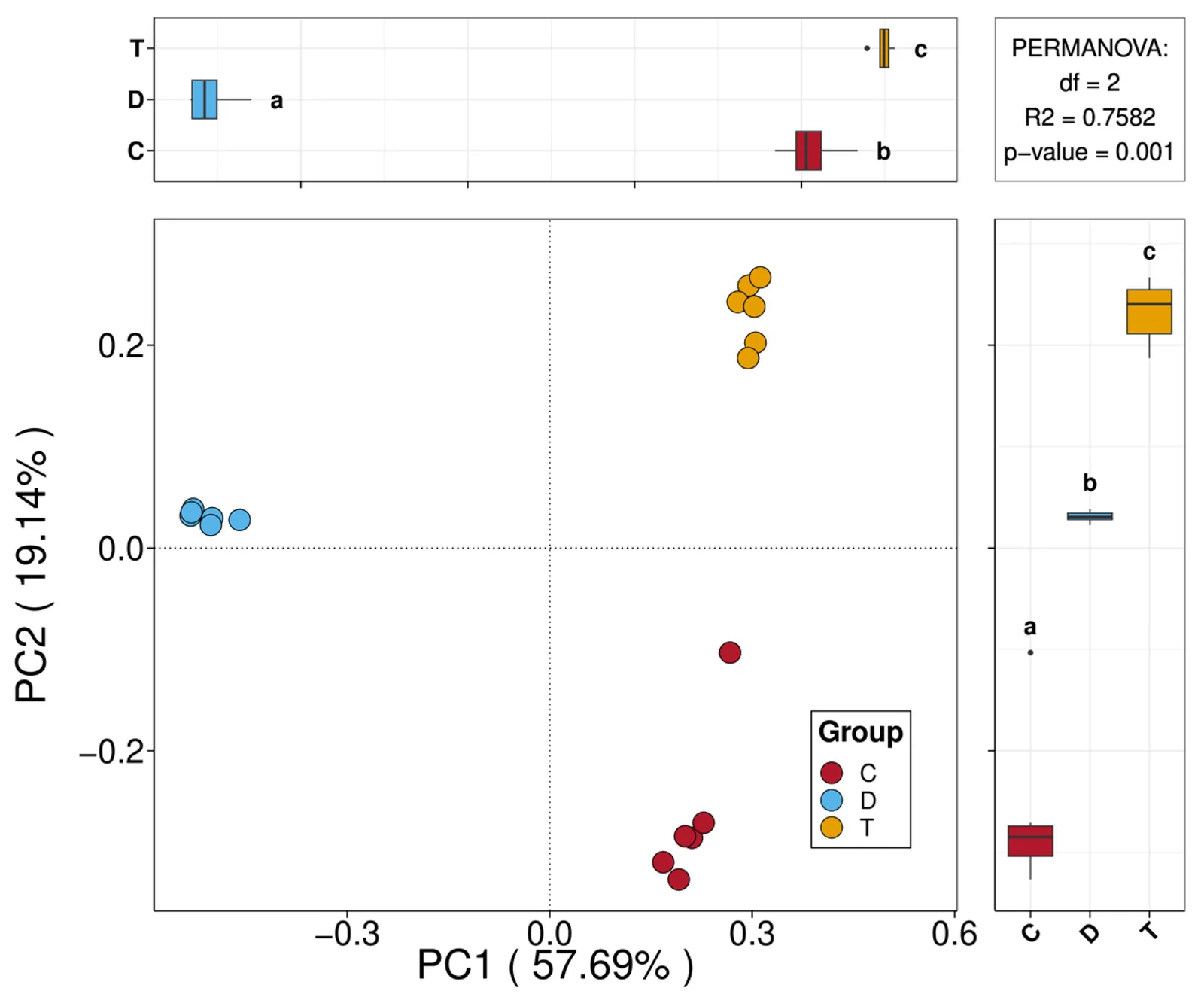
PCoA analysis based on Bray–Curtis algorithm.

**Figure 5:**
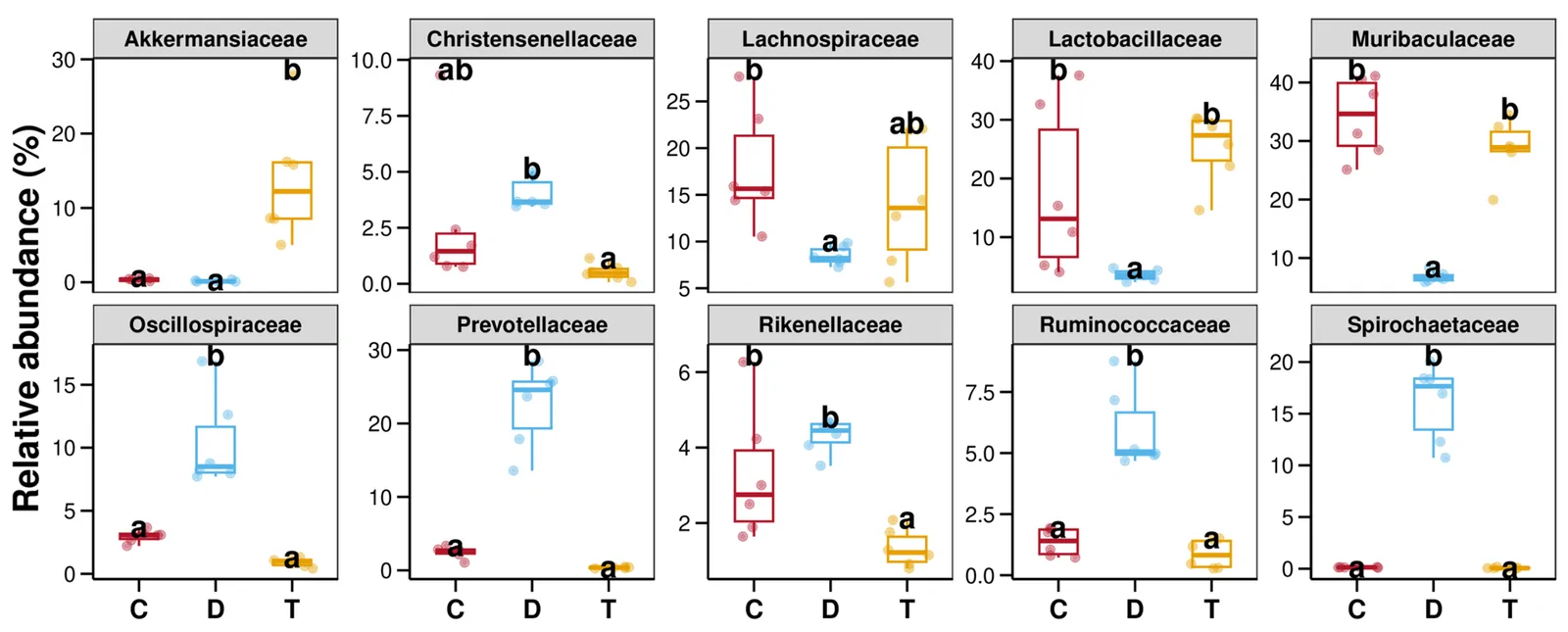
Top 10 differential families in relative abundance among groups. Different lowercase letters denote statistically significant differences between groups (*p* < 0.05); identical letters indicate no significant differences. C = control mice, D = sika deer donor, and T = FMT-transplanted mice.

**Figure 6:**
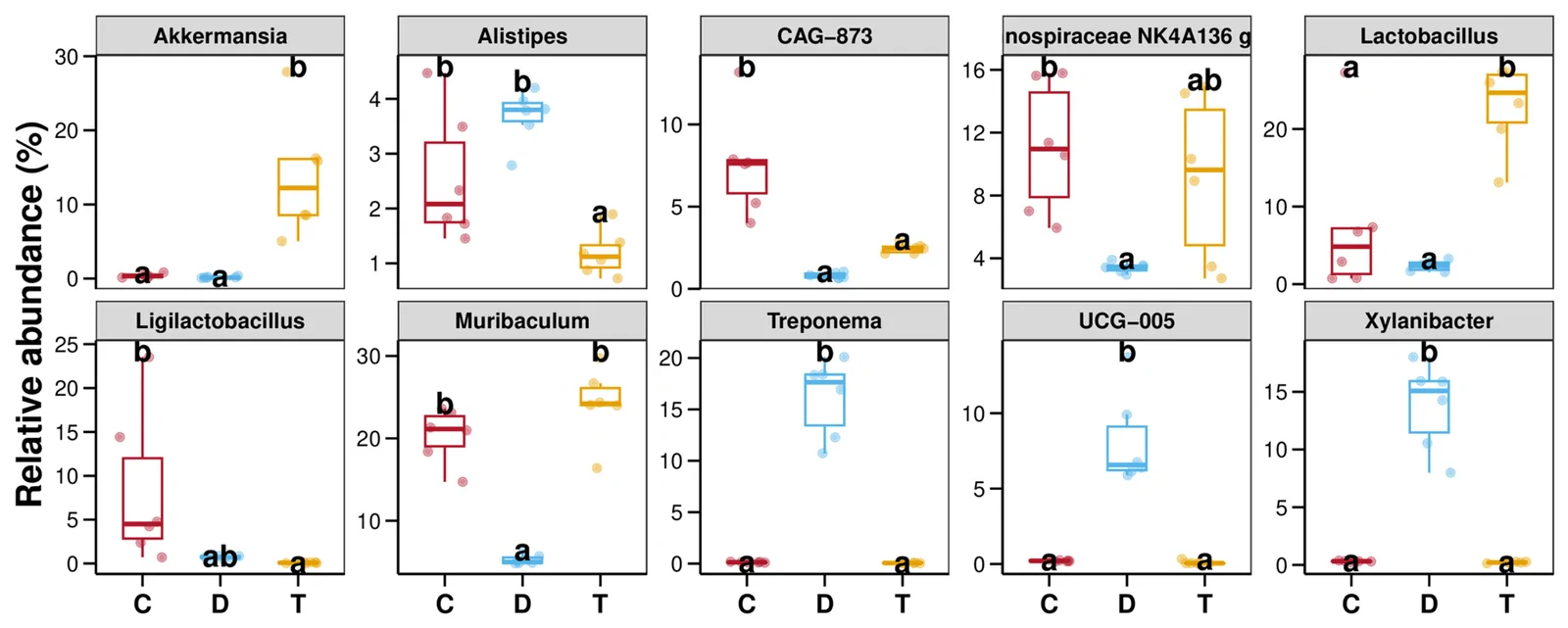
Top 10 differential genera in relative abundance among groups. Different lowercase letters denote statistically significant differences between groups (*p* < 0.05); identical letters indicate no significant differences. C = control mice, D = sika deer donor, and T = FMT-transplanted mice.

**Figure 7:**
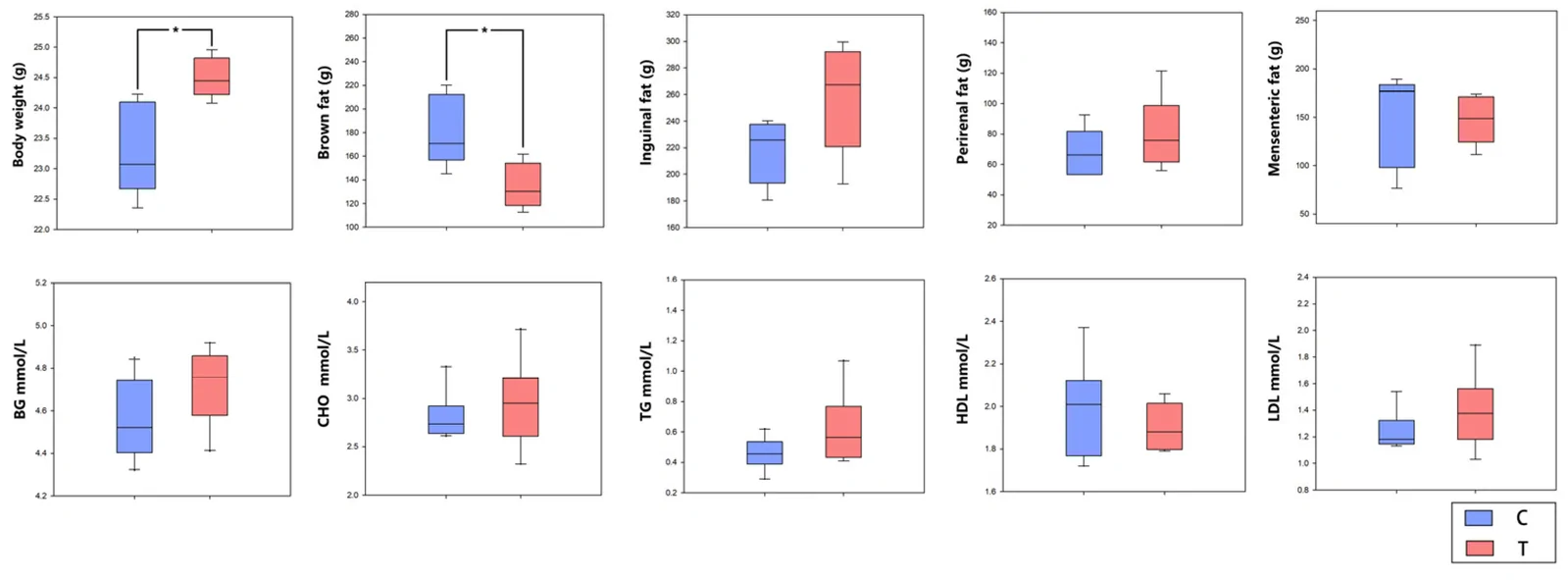
Serum biochemical indices and fat deposition parameters in mice (Mean ± SD, *n* = 5). Boxplots showing body weight, brown fat weight, inguinal fat weight, perirenal fat weight, mesenteric fat weight, blood glucose (BG), total cholesterol (CHO), triglycerides (TGs), high-density lipoprotein (HDL), and low-density lipoprotein (LDL) in the control group (C) and FMT group (T). * *p* < 0.05. Individual raw data points are shown in Figure S2.

**Figure 8:**
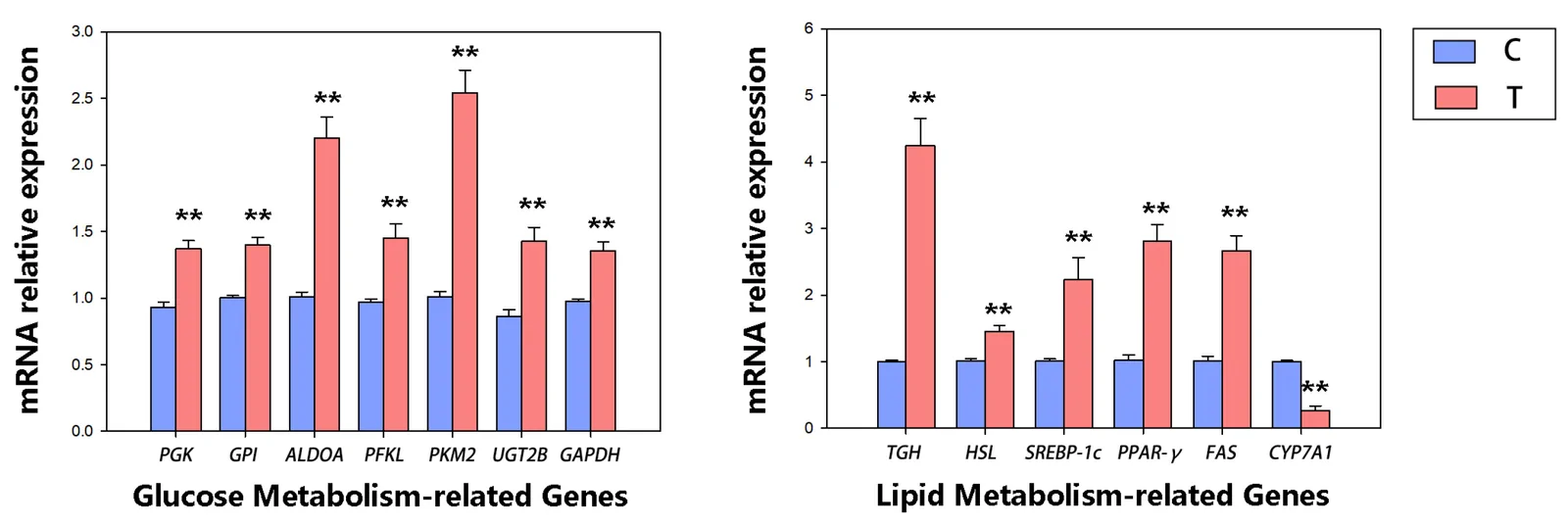
Hepatic relative mRNA expression of glucose and lipid metabolism-related genes in control (C) and transplanted (T) groups. Bars represent the mean, and error bars indicate the standard error of the mean (SEM). C, control group; T, transplanted group. ** *p* < 0.01. Individual raw data points are shown in Figure S3.

**Figure 9:**
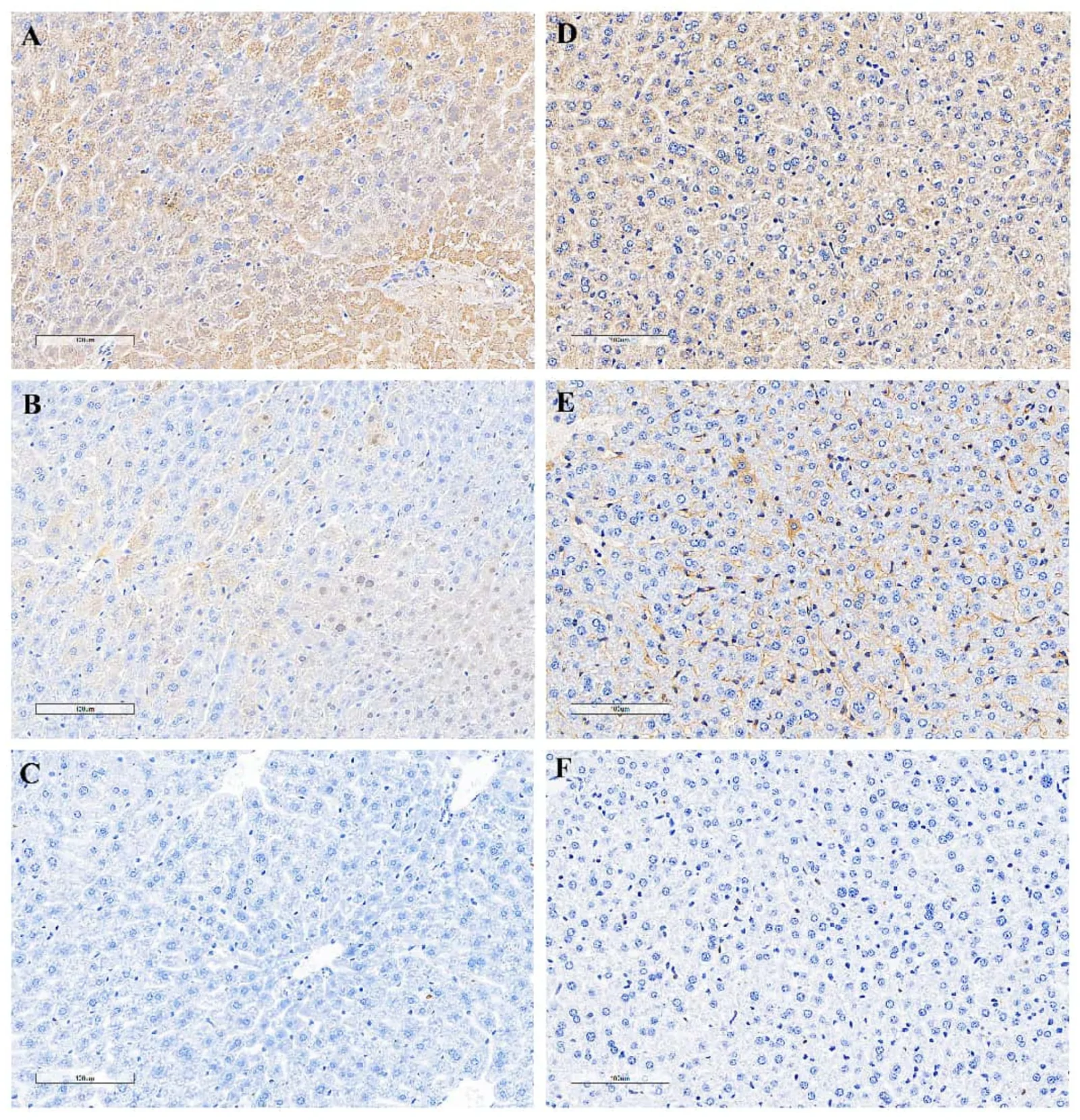
Hepatic immunohistochemical staining of target proteins in mice. IHC images of liver tissues are displayed in panels A–F (20× objective, scale bar = 100 μm). (**A**–**C**) Transplanted group; (**D**–**F**) control group. (**A**,**D**) CYP7A1; (**B**,**E**) PPARγ; (**C**,**F**) PFKL. Positive protein signals were visualized as brown staining in hepatocytes. Compared with the control group, the transplanted group exhibited significantly higher PPARγ expression, decreased CYP7A1 expression, and elevated PFKL expression.

**Figure 10:**
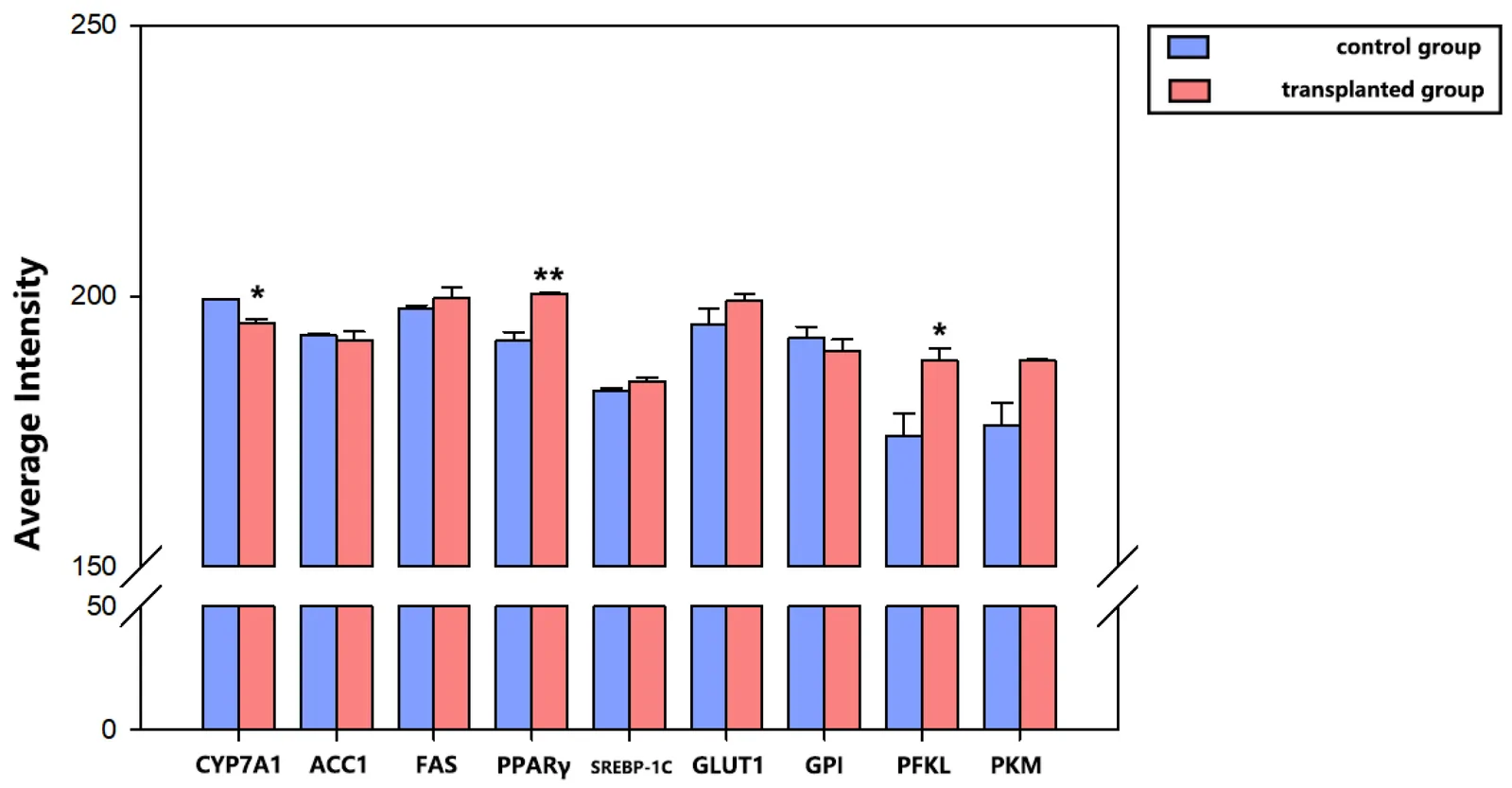
Average optical density of hepatic immunohistochemical staining in mice. Bars represent the mean, and error bars indicate the standard error of the mean (SEM). C, control group; T, transplanted group. * *p* < 0.05, ** *p* < 0.01.

## Data Availability

The raw data supporting the conclusions of this article will be made available by the authors on request.

## Supplementary Materials

The following supporting information can be downloaded at: https://www.mdpi.com/article/10.3390/vetsci13101076/s1, Table S1: Primers used in this study; Table S2: Antibodies used in this study; Figure S1: Permutation test histogram (1000 permutations) of the OPLS‑DA model; Figure S2: Serum biochemical indices and fat deposition parameters in mice (Mean ± SD, *n* = 5); Figure S3: Hepatic RNA relative expression of glucose and lipid metabolism-related genes in control (C) and transplanted (T) groups; Figure S4: Average optical density of hepatic immunohistochemical staining in mice.
