# Supplementary material for: Study on Intestinal Adaptation Mechanism of Sika Deer to High-Tannin Diet and the Mediating Role of Gut Microbiota
Source: Vet Sci. 2026 Oct 12;13(10):1076. doi: 10.3390/vetsci13101076 (PMC13709731; doi:10.3390/vetsci13101076)
Supplement: Supplementary file 1 [file vetsci-13-01076-s001.zip › vetsci-4602034-supplementary.pdf]

veterinary sciences

MDPI

Supplementary Materials

Study on Intestinal Adaptation Mechanism of Sika Deer to High-Tannin Diet and the Mediating Role of Gut Microbiota

Tianxiang Zhang [1,2,†], Yang Zhang [1,2,†], Manyu Zhang [1,2], Jun Liu [1,2], Yixuan Zhang [1,2] ,

Yingying Feng [1,2], Weijie Han [1,2], Ziwei Zhu [1,2], Haijun Chen [3], Xiaofeng Huang [1,2,*].

1 Jiangxi Academy of Forestry, Nanchang 330032, China
2 Key Laboratory of Wildlife Conservation, National Forestry and Grassland Administration (Jiangxi Academy of Forestry), Nanchang 330032, China
3 Xiajiang Lishan Sika Deer Breeding Base, Ji'an 331401, China
* Correspondence: huangxiaofeng0791@126.com
† These two authors contributed equally to this work.

Supplementary Tables

Table S1. Primers used in this study

Gene	NCBI RefSeq accession number	Primer 5′-3′
β-actin	NM_007393.5	F: AGGGAAATCGTGCGTGAC
		R: CATACCCAAGAAGGAAGGCT
GPI	NM_008155.4	F: TGACTGGAAAGGGTACACTGG
		R: TGGGTCCCATCAATGTTAGAGAC
PFKL	NM_008826.5	F: GAACTACGCACACTTGACCAT
		R: CTCCAAAACAAAGGTCCTCTGG
ALDOA	NM_007438.5	F: AGTCCACCGGAAGCATTGC
		R: CAGCCCCTGGGTAGTTGTC
PGK	NM_008828.3	F: ATGTCGCTTTCCAACAAGCTG
		R: GCTCCATTGTCCAAGCAGAAT
UGT2B	NM_001029867.1	F: TTGATGTCGTTCTAGCAGATGC
		R: TCAAAAAGCAACACCTGCAAC
PKM2	NM_011099.4	F: TTCGCATGCAGCACCTGATT
		R: AGCAGTCTGGGGATTTCGAG
GAPDH	NM_008084.4	F: TCAACGGCACAGTCAAGG
		R: TGAGCCCTTCCACGATG
HK2	NM_013820.4	F: CCACTCCAGACGGTACAGAGAA
		R: CCTTCGCTTGCCATTACGC
TGH	NM_053200.2	F: CTTGGCTCCTTGAGATTTGCT
		R: CCATCACTGGTAGTCTGCTGT
HSL	NM_001039507.2	F: GATTTACGCACGATGACACAGT
		R: ACCTGCAAAGACATTAGACAGC
CYP7A1	NM_007824.3	F: GCTGTGGTAGTGAGCTGTTG
		R: GTTGTCCAAAGGAGGTTCACC
SREBP-1c	NM_011480.4	F: AGGATAGCCAGGTCAAAGCC
		R: ATGCCCCAGCCGAAAAG
FAS	NM_007987.2	F: TGCGATGAAGAGCATGGTTTAGA
		R: GGTCAACAACCATAGGCGATTTC
PPARγ	NM_001308354.2	F: GCCCACCAACTTCGGAATC
		R: GCAGAAATGCTGGAGAAATCAA

Table S2. Antibodies used in this study

Antigen	Antibody	Uniprot ID	Art. No.	Company
CYP7A1	Recombinant Anti-CYP7A1 antibody (Mouse mAb)	P22680	GB150112-100	Servicebio
ACC1	Recombinant Anti-Acetyl-CoA Carboxylase antibody (Rabbit mAb)	Q13085	GB150064-100	Servicebio
FAS	Anti-Fas Mouse mAb	P25446	GB12089-100	Servicebio
PPARγ	Anti-PPAR gamma Rabbit pAb	P37238	GB11164-100	Servicebio
SREBP-1c	Anti-SREBP1 Rabbit pAb	Q9WTN3	GB11524-100	Servicebio
GLUT1	Recombinant Anti-Glucose Transporter GLUT1 antibody (Rabbit mAb)	P11166	GB153495-100	Servicebio
GPI	Anti-AMF Rabbit pAb	P06745	GB113691-100	Servicebio
PFKL	Anti-PFKL Rabbit pAb	P17858	GB111136-100	Servicebio
PKM	Recombinant Anti-PKM2 antibody (Mouse mAb)	P14618	GB15392-100	Servicebio

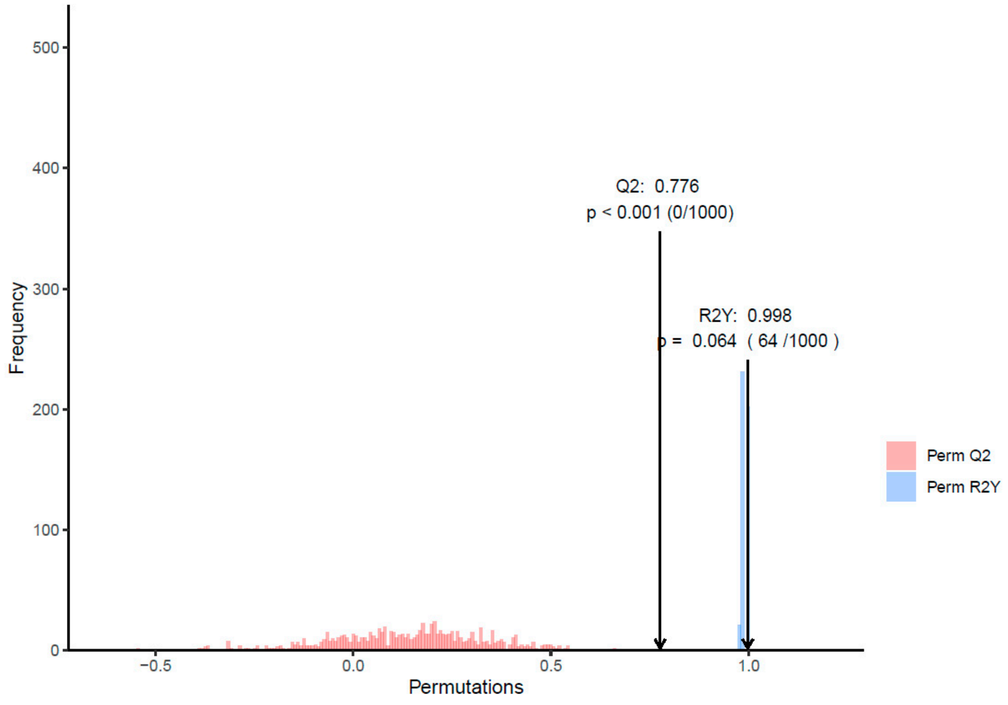

Figure S1. Permutation test histogram (1000 permutations) of the OPLS-DA model

The vertical black lines represent the R^2Y (0.998) and Q^2 (0.776) values of the original OPLS-DA model. The blue and red histograms show the distribution of R^2Y and Q^2 values obtained from 1000 permutation tests, respectively. P<0.001 for Q^2.

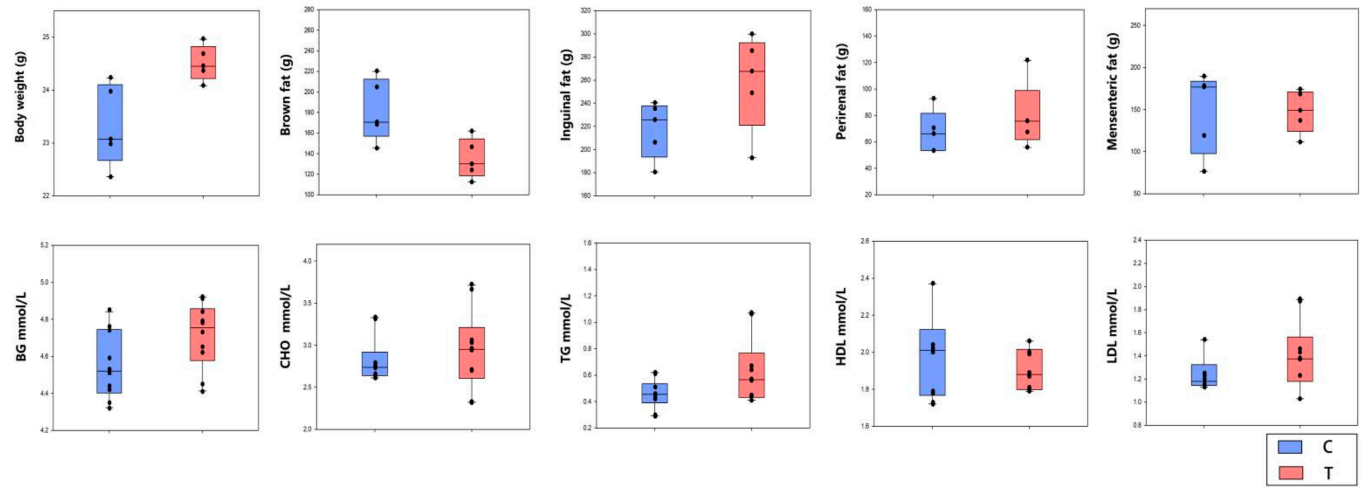

Figure S2. Serum biochemical indices and fat deposition parameters in mice (Mean ± SD, n = 5)

Boxplots overlaid with individual data points showing body weight, brown fat weight, inguinal fat weight, perirenal fat weight, mesenteric fat weight, blood glucose (BG), total cholesterol (CHO), triglyceride (TG), high-density lipoprotein (HDL), and low-density lipoprotein (LDL) in the control group (C) and microbiota-transplanted group (T). Each dot represents an individual sample.

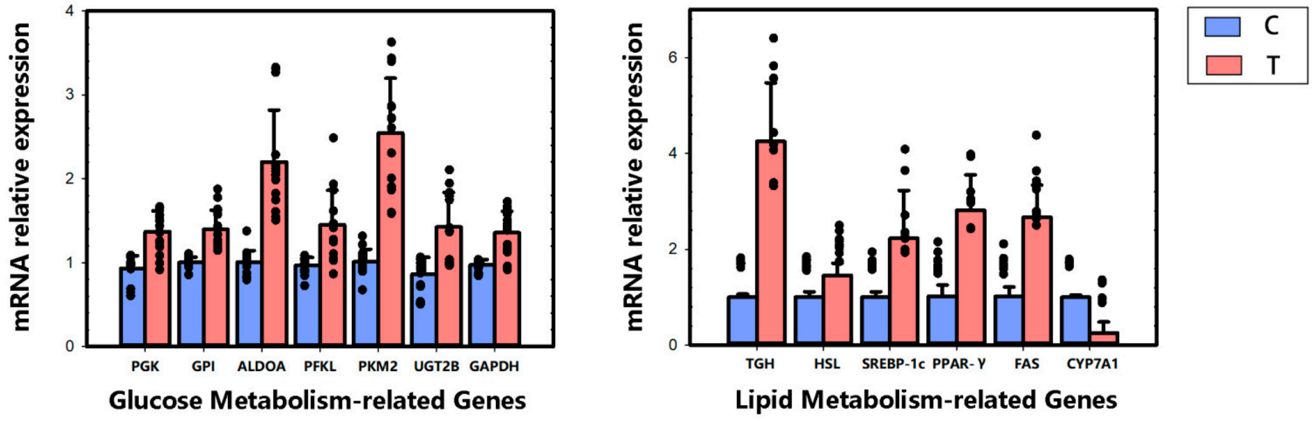

Figure S3. Hepatic RNA relative expression of glucose and lipid metabolism-related genes in control (C) and transplanted (T) groups

Bar charts overlaid with individual data points showing relative mRNA expression levels of glucose metabolism-related genes and lipid metabolism-related genes in liver tissues from control group (C) and transplanted group (T). Each dot represents an individual sample.

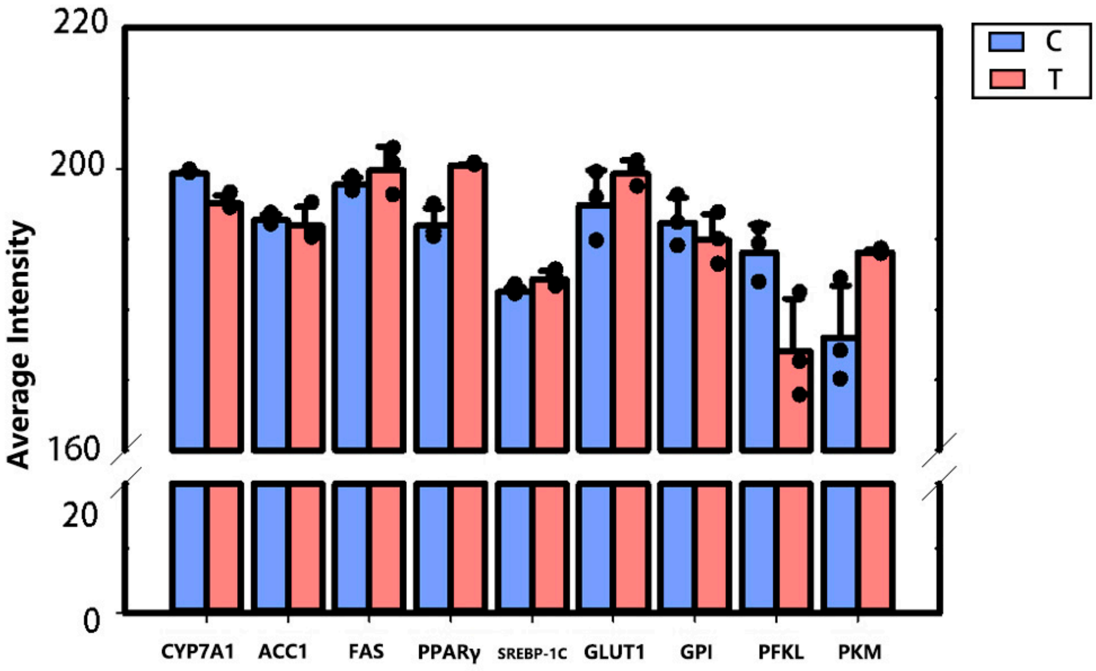

Figure S4. Average optical density of hepatic immunohistochemical staining in mice

Bar charts overlaid with individual data points showing the average immunohistochemical staining intensity of metabolism-related proteins in liver sections of control group (C) and transplanted group (T). Each dot represents the averaged intensity calculated from one of three different imaging regions within a single liver tissue section.
